# Association between level of compliance with COVID-19 public health measures and depressive symptoms: A cross-sectional survey of young adults in Canada and France

**DOI:** 10.1371/journal.pone.0289547

**Published:** 2023-08-02

**Authors:** Pierre-julien Coulaud, Julie Jesson, Naseeb Bolduc, Olivier Ferlatte, Karine Bertrand, Travis Salway, Marie Jauffret-Roustide, Rod Knight

**Affiliations:** 1 British Columbia Centre on Substance Use, Vancouver, British Columbia, Canada; 2 Department of Medicine, University of British Columbia, Vancouver, British Columbia, Canada; 3 Faculty of Health Sciences, Simon Fraser University, Burnaby, British Columbia, Canada; 4 School of Population and Public Health, University of British Columbia, Vancouver, British Columbia, Canada; 5 Département de Médecine Sociale et Préventive, École de Santé Publique de l’Université de Montréal, Montréal, Québec, Canada; 6 Centre de Recherche en Santé Publique, Université de Montréal et CIUSSS du Centre-Sud-de-l’Île-de-Montréal, Montréal, Canada; 7 Faculté de Médecine et des Sciences de la Santé, Université de Sherbrooke, Longueuil, Québec, Canada; 8 British Columbia Centre for Disease Control, Vancouver, British Columbia, Canada; 9 Centre for Gender and Sexual Health Equity, Vancouver, British Columbia, Canada; 10 Centre d’Étude des Mouvements Sociaux (EHESS/CNRS UMR8044/INSERM U1276), Paris, France; 11 Baldy Center on Law and Social Policy, Buffalo University, Buffalo, NY, United States of America; Friedrich-Alexander-Universität Erlangen-Nürnberg: Friedrich-Alexander-Universitat Erlangen-Nurnberg, GERMANY

## Abstract

**Background:**

While compliance with preventive measures remains central to limit the spread of COVID-19, these measures critically affected mental health of young adults. We therefore investigated the association between the level of compliance with COVID-19 preventive measures and depressive symptoms among young adults in Canada and France.

**Methods:**

From October to December 2020, we conducted a cross-sectional online survey of young adults ages 18–29 years in Canada (n = 3246) and France (n = 2680) to collect demographic data, experiences with COVID-19 preventive measures, and mental health. Depressive symptoms were assessed by the Patient Health Questionnaire-9 (PHQ-9). Compliance profiles were built using cluster analysis. Weighted multivariable logistic regression was used to estimate associations between compliance level and major depressive symptoms (PHQ-9 score≥15) in each country.

**Results:**

One third of respondents reported major depressive symptoms (Canada: 36.4%, France: 23.4%). Four compliance profiles were identified: high (42.5%), medium-high (21.7%), medium-low (18.1%), and low (17.7%), with high levels more frequently observed in Canada compared to France. In both countries, participants in low compliance profile (Canada: Adjusted Odds Ratio (AOR) [95% Confidence Interval] 0.75 [0.58, 0.98], France: AOR 0.60 [0.46, 0.75]), in the medium-low (Canada: AOR 0.58 [0.48, 0.72], France: AOR 0.81 [0.66, 1.01]), and medium-high compliance profiles (Canada: AOR 0.78 [0.65, 0.93], France: AOR 0.77 [0.63, 0.93]) were less likely to report major depressive symptoms compared to the high compliance profile. Ethno-racial minorities, sexual and gender minority, and unemployed young adults had higher odds of reporting such symptoms.

**Conclusions:**

Major depressive symptoms were associated with high compliance with COVID-19 preventive measures among young adults. The implementation of socially-isolating measures should be coupled with mental health interventions to address mental health needs of young adults, with enhanced supports for sub-groups who are structurally disadvantaged (e.g., racialized, unemployed, sexual and gender minority).

## Introduction

Since the beginning of the COVID-19 pandemic, various jurisdictions around the world implemented public health preventive measures to prevent the spread of the virus [1]. Depending on the jurisdiction and the level of COVID-19 severity, preventive measures have featured educational guidance (e.g., hygiene recommendations, like frequent handwashing), as well as enforceable public health orders such as bans on social gatherings, closures of schools and non-essential businesses, stay-at-home orders and the use of face masks [2–4]. Throughout each successive wave of COVID-19, modelling of public compliance with preventive measures suggests the potential for a decrease in the onward spread of the virus and a corresponding flattening of the COVID-19 curve—evidence used by public health officials to justify prevention education and restrictive orders [5, 6].

Preliminary evidence collected in the first months of the COVID-19 pandemic (March-August 2020) highlights that young adults (<30 years) had lower levels of engagement in some preventive behaviors, including physical distancing and hygiene measures, than older adults [7–11]. Previous research has also identified that COVID-19 preventive measures that require varying degrees of social isolation may be particularly harmful for the mental health and well-being of young adult populations [12–15]. Findings from longitudinal studies examining the mental health impacts of COVID-19 on the general population further indicate that young adults (e.g., 18–29 years) were more likely to report higher levels of depressive and anxiety symptoms and suicide ideation compared to older age groups [16–18]. As such, there is concern that the psychosocial needs of young adults were not sufficiently addressed during various phases of the pandemic [19].

To date, research examining the association between depression and compliance with COVID-19 preventive measures has been mainly conducted among adults, and these results are equivocal. For example, several studies showed that adherence to distancing behaviors and staying at home was associated with increased risk for depression [20–23] while other studies reported no significant association [24–26] or that being more compliant was a protective factor for depressive symptoms [7, 27, 28]. In addition, most of these studies only assessed a limited number of preventive measures that were investigated separately for effects on depressive symptoms, leaving questions as to the extent to which complying with multiple concurrent COVID-19 preventive measures may have an additive effect on depressive symptoms.

The objective of this study was therefore to investigate the association between the level of compliance with multiple COVID-19 preventive measures and depressive symptoms among young adults living in Canada and France. We hypothesized that we would observe a significant association between the levels of compliance with COVID-19 preventive measures and depressive symptoms, such that those who report lower levels of compliance will experience lower levels of depressive symptoms. Our hypothesis is based on the assumptions that young adults who were highly compliant to socially restrictive COVID-19 measures would report fewer opportunities to connect with family/friends. As such, they would be less likely to receive and benefit from social supports, which would lead to an increased risk of experiencing depressive symptoms.

## Materials and methods

### Study design and settings

Data were collected through the *France-Canada Observatory on COVID-19*, *Youth health and Social well-being (FOCUS*) study, a cross-sectional, online anonymous survey exploring the impact of the pandemic on social and health outcomes among young adults ages 18–29 years in Canada and France. The survey was conducted from the 8^th^ of October to the 23^rd^ of December 2020.

Canada and France are two high-income countries that have been particularly affected by the COVID-19 pandemic, respectively with 3.8 and 28.8 million total number of cases since March 2020 [29]. In both countries, young adults have experienced high rates of COVID-19 infection (compared to older adults groups), comprised 19% of total cases in Canada [30] and 17% in France [31].

In Canada, the first peak of COVID-19 cases started in mid-March 2020 with a major outbreak in the province of Quebec [32]. Between March and June 2020, the federal and provincial governments implemented a series of public health measures (e.g., stay-at-home orders, closure of schools and pivot to online course delivery) to limit the spread of the pandemic [33]. The second peak was reached in early January 2021. In October-November 2020, each province and territory introduced measures from avoiding non-essential travel and limiting social gatherings to the closure of non-essential businesses. At the end of December 2020, province-wide lockdowns were announced in Quebec and Ontario [34, 35].

In France, the COVID-19 pandemic spiked dramatically in March-April 2020, mainly in the northeast and the region of Paris. Between March and May 2020, a national lockdown was implemented to control the spread of the pandemic before a gradual reopening of activities and businesses over the summer [36]. The peak of the second wave was reached in early November 2020. The Government of France implemented an increasing series of mitigation measures in late September (e.g., limiting social gatherings, closing bar/restaurants) and throughout October, including a curfew in metropolitan areas (October 17–30), a nationwide lockdown (October 30-December 15), and a national curfew that began 15 December 2020 [36, 37]. During this period, schools and universities remained mainly open under certain conditions (e.g., capacity limits, mask mandate) to limit social inequalities.

### Participants and procedures

Survey participants were recruited through non-probability sampling using online posts and advertisements on social media platforms (e.g., Facebook and Instagram) and university websites, and word of mouth. Additional efforts were made to reach underrepresented populations and geographic areas by using targeted ads from demographic criteria available on social media platforms (e.g., age, gender). Participants were eligible if they were: (1) between the age of majority (18 or 19 in Canada depending on the province or territory of residence; 18 in France) and 29 years; resided in Canada or France; and (2) able to complete the survey in English (Canada) or French (either country). The questionnaire comprised four major sections: sociodemographic, social life and experiences during the pandemic (including questions about COVID-19 preventive measures), access to social and health services, and health outcomes (e.g., mental health). In order to ascertain the acceptability of the questionnaire, we invited 10 youth (n = 5 in each country) to pre-test the questionnaires and provide insights about the format, content and sequencing of the questionnaire, including whether questions were clear and the responses available for each question were appropriate. Survey data were collected using *Qualtrics*. On the first page of the online survey, all participants were provided with the study’s objective and details about how to participate. Prior to accessing the online questionnaire, all participants were informed that the completion of the questionnaire implied informed consent. Participants were also informed that they could stop the survey questionnaire at any time. All participants were also provided with an option to enter a draw to win one of three cash prizes (CAD$100 in Canada, 100€ in France). Ethical approval was granted by the University of British Columbia Behavioural Research Ethics Board (H20-02053).

To ensure survey security and protect our online survey from fraudulent submissions (including duplicate submissions and “bot” attacks), we used several preventive methods including setting up security survey options in our survey tool *Qualtrics* to access our online questionnaire (e.g., procedures to detect fake IP address), screening time and duration of survey completion, and checking for inconsistencies across each new submission [38]. Furthermore, our survey incentives were relatively small for participants (i.e., a lottery draw to win one of three cash prizes), and our survey promotion activities did not include information about incentives, procedures that are known to reduce duplicate responses and fraudulent submissions [39].

### Study population

The population for the present study included participants of the FOCUS study who had complete data on socio-demographics, COVID-19 preventive measures, and mental health questions. Therefore, participants who had missing information on socio-demographics, the questions about the COVID-19 preventive measures or did not complete the depression scale were excluded.

### Outcome

Depressive symptoms within the past two weeks were measured using the Patient Health Questionnaire-9 (PHQ-9) [40], a validated scale that has been translated in multiple languages, including French [41]. Because a previous study demonstrated that the English and French PHQ-9 versions did not present substantial differences in scoring metrics [42], we did not perform psychometric analyses to examine the effect of the different languages on depression scores. Each of the 9 items are scores from 0 (not at all) to 3 (nearly every day) with total score ranges from 0 to 27. Scores within 0–4 are considered as having minimal, 5–9 mild depressive symptoms, 10–14 moderate depressive symptoms, 15–19 moderately severe depressive symptoms and 20–27 severe depressive symptoms [43]. To limit overestimation of the prevalence of depression in our study, the higher cut-off score of 15 was used to identify participants with major depressive symptoms [44].

### Exposure

Our primary exposure was the level of compliance with COVID-19 preventive measures. Using a multiple-choice question with ten response options, we asked participants whether they took any actions to decrease their risk of getting or transmitting COVID-19 in the past 6 months. The following five socially-isolating preventive measures were used to build a COVID-19 compliance profiles variable (see ‘Statistical analysis’ section): staying home from work or school, only taking essential trips, avoiding social gatherings of over 10 people, avoiding meeting friends, and maintaining a social bubble at home. Five other preventive measures were collected including health behaviors (practicing physical distancing, wearing a face covering), and hygiene practices (washing hands, cleaning frequently touched surfaces and objects, avoiding touching face with unwashed hands); however, we *a priori* determined that these measures were not conceptually linked to mental health in the same manner as the socially-isolating measures and therefore were not included in the construction of compliance profiles.

### Covariates

The covariates included the following socio-demographic characteristics: age, gender identity, sexual orientation, province/region of residence, area of residence (large urban centre 100,000+ people versus medium or small city), highest degree of education, employment status, and living arrangements. In Canada, ethno-racial identity was collected using the Canadian Institute for Health Information standards [45]. In France, where asking about ethno-racial identity is not permitted, participants were asked to provide the country of birth of their parents, and maternal and paternal grand-parents. French participants who reported that at least one of their parents or two of their grandparents from the same side were born outside France or Europe were considered as descendants of immigrants [46].

To best capture the climate of uncertainty regarding the evolution of the COVID-19 pandemic and the economic downturn that young adults experienced at the time of the survey (e.g., COVID-19 vaccines were not yet available in Fall 2020), we included four other covariates in our analysis. Level of concern for COVID-19’s impact was assessed by asking the following questions: “How concerned are you about the impact of COVID-19 on: (1) the economy and businesses; and (2) the uncertainty of the future”. Response options were classified in low (not or a bit concerned) and high level of concern (quite or very concerned). We also asked participants if they have: (3) been tested for COVID-19 in the past 6 months (yes versus no), and (4) lost any individual income (including salary, employment insurance, government assistance, *etc*.) due to the COVID-19 pandemic. Given the limited number of participants who reported a positive test for COVID-19 (e.g., n = 22, 0.7% in Canada, n = 135, 5% in France), we did not include the results of the tests in our analysis.

All of these covariates were considered as potential confounders in our analysis because: a) they are empirically demonstrated explanatory factors (social determinants) associated with depression [47–49], and b) are empirically or conceptually related to our exposure variable of compliance with COVID-19 preventive measures [50–52].

### Statistical analyses

Using data from both countries, we conducted a cluster analysis to identify COVID-19 compliance profiles. The K-means method of cluster analysis was applied to classify participants into mutually exclusive groups, using Euclidean distances with cluster centers based on least squares estimation (SAS PROC FASTCLUS procedure) [53]. The number of clusters was determined by using the pseudo-F statistic, approximate expected overall R square, and cubic clustering criterion, as well as by considering the most meaningful clusters regarding the compliance to COVID-19 preventive measures. A descriptive analysis was then performed to present the characteristics of each profile in both countries (see S1 Table).

Unadjusted and adjusted associations between major depressive symptoms (PHQ-9 score ≥15) and level of compliance were estimated using multivariable logistic regression, adjusted for all covariates listed above. In our models, participants with high compliance were considered as the reference group because both Canadian and French health authorities recommended at the time of the survey to comply with the COVID-19 preventive measures included in our profile construction (e.g., avoiding meeting friends and social gatherings). In our multivariate logistic regression models (see Table 2), we use listwise deletion to remove participants with missing data across covariates and those who selected the “Prefer not to say” option responses, as they represented a limited number of participants (5% of the total sample). We applied survey weights by age, gender, and province/region of residence using official census data in each country to improve the representativeness of the sample (see here [54] for further details). We also applied a statistical test (i.e., the Fairchild test) to examine how the magnitude of the association between depression and COVID-19 compliance differed between Canada and France (see S2 Table) [55]. All analyses were performed using R version 4.0.3 and SAS University software (SAS Institute Inc., Cary, NC, USA).

## Results

Of the 8424 participants of the FOCUS survey, 89.6% completed the initial sociodemographic information. Of these, we excluded those who did not respond to the COVID-19 preventive measures (n = 383), and mental health questions (n = 1286), resulting in a final study sample of 5926 (Canada: n = 3246, 54.8% and France: n = 2680, 45.2%). Participants excluded (n = 1619, 27.3%) were more likely to come from France (61.1% vs. 38.9%), be younger (18–19 years; 26.9% vs. 17.9%), identify as men (38.4% vs. 29.5%) and as straight/heterosexual (65.5% vs. 59.5%), live in medium or small cities (53.5% vs. 46.5%), report a lower education level (high school or college; 50.7% vs. 38.2%) compared to the participants included in this analysis (see S3 Table). Participant characteristics in both countries are described in Table 1. Significant differences were observed between the two study samples in the main sociodemographic characteristics. Compared to the French sample, participants in Canada were more likely to identify as a sexual minority (e.g., bisexual: 19% vs. 12.9%), live in large urban centres (60.5% vs. 51%), and report a lower education level (e.g., university graduate degree: 10% vs. 29.1%). In Canada, young adults were more likely to be employed (40.8% vs. 35% in France) or student-employed (25.7% vs. 17% in France) while participants in France were more likely to be students (35.3% vs. 21.7% in Canada) and report living alone (31.9% vs. 14% in Canada). Overall, one third of the total sample (30.5%) reported major depressive symptoms with a higher prevalence in Canada (36.4%) compared to France (23.4%).

**Table 1:** Characteristics of the study population, total and by country.

		Weighted, n (column %)		
		Total	Canada	France
**All participants**		5926 (100)	3246 (54.8)	2680 (45.2)
**Socio-demographic characteristics**				
Age (years)				
	18–19	960 (16.2)	501 (15.4)	459 (17.1)
	20–24	2480 (41.8)	1379 (42.5)	1101 (41.1)
	25–29	2486 (42)	1366 (42.1)	1120 (41.8)
Gender identity				
	Man	2868 (48.4)	1547 (47.7)	1321 (49.3)
	Woman	2649 (44.7)	1411 (43.5)	1238 (46.2)
	Non-binary/other gender identity^$^	409 (6.9)	288 (8.9)	121 (4.5)
Sexual orientation				
	Straight/heterosexual	3600 (60.7)	1817 (56)	1783 (66.5)
	Bisexual	962 (16.2)	617 (19)	345 (12.9)
	Other sexual minorities^£^	1211 (20.4)	733 (22.6)	478 (17.8)
	Prefer not to say	151 (2.5)	78 (2.4)	73 (2.7)
Ethno-racial identity (only in Canada)^§^				
	Non-racialized	2821 (47.6)	2821 (86.9)	_
	Indigenous	149 (2.5)	149 (4.6)	_
	Racialized, non-Indigenous	276 (4.7)	276 (8.5)	_
Descendants of immigrants (only in France)				
	No	2307 (38.9)	_	2307 (86.1)
	Yes	353 (6)	_	353 (13.2)
	Prefer not to say	20 (0.3)	_	20 (0.7)
Province or territory of residence (Canada)^¶^				
	Ontario	1174 (19.8)	1174 (36.2)	_
	Atlantic	204 (3.4)	204 (6.3)	_
	British Columbia/Territories	472 (8)	472 (14.5)	_
	Prairies	659 (11.1)	659 (20.3)	_
	Quebec	737 (12.4)	737 (22.7)	_
Regions of residence (France)^^^				
	Ile-de-France	658 (11.1)	_	658 (24.6)
	Nord-Est	529 (8.9)	_	529 (19.7)
	Ouest	450 (7.6)	_	450 (16.8)
	Outre-mer	76 (1.3)	_	76 (2.8)
	Sud-Est	542 (9.1)	_	542 (20.2)
	Sud-Ouest	426 (7.2)	_	426 (15.9)
Area of residence				
	Large urban centre	3331 (56.2)	1963 (60.5)	1368 (51)
	Medium or small city	2595 (43.8)	1283 (39.5)	1312 (49)
Highest level of education				
	High school college	2209 (37.3)	1285 (39.6)	924 (34.5)
	Some university	2595 (43.8)	1628 (50.2)	967 (36.1)
	University graduate degree	1105 (18.6)	326 (10)	779 (29.1)
	Missing data	17 (0.3)	7 (0.2)	10 (0.4)
Employment status				
	Employed	2262 (38.2)	1323 (40.8)	939 (35)
	Student	1651 (27.9)	706 (21.7)	945 (35.3)
	Student and employed	1278 (21.6)	823 (25.4)	455 (17)
	Unemployed	687 (11.6)	361 (11.1)	326 (12.2)
	Missing data	47 (0.8)	33 (1)	14 (0.5)
Living arrangements				
	Alone	1309 (22.1)	455 (14)	854 (31.9)
	With family members	1973 (33.3)	1164 (35.9)	809 (30.2)
	With partner	1445 (24.4)	845 (26)	600 (22.4)
	With roomate/friends/other	1196 (20.2)	782 (24.1)	414 (15.4)
	Missing data	3 (0.1)	_	3 (0.1)
**COVID-19-related concerns and experiences**				
Level of concern about the uncertainty of the future				
	Low	1531 (25.8)	844 (26)	687 (25.6)
	High	4364 (73.6)	2386 (73.5)	1978 (73.8)
	I don’t know	28 (0.5)	16 (0.5)	12 (0.4)
	Missing data	4 (0.1)	_	4 (0.1)
Level of concern for economy				
	Low	2340 (39.5)	1360 (41.9)	980 (36.6)
	High	3543 (59.8)	1857 (57.2)	1686 (62.9)
	I don’t know	41 (0.7)	29 (0.9)	12 (0.4)
	Missing data	2 (0)	_	2 (0.1)
Being tested for COVID-19				
	No	3659 (61.7)	2112 (65.1)	1547 (57.7)
	Yes	2248 (37.9)	1124 (34.6)	1124 (41.9)
	Missing data	18 (0.3)	9 (0.3)	9 (0.3)
Income loss				
	No	3612 (61)	1610 (49.6)	2002 (74.7)
	Yes	2314 (39)	1636 (50.4)	678 (25.3)
**Mental health**				
Depressive symptoms (PHQ-9)				
	Mild	1719 (29)	853 (26.3)	866 (32.3)
	Minimal	1084 (18.3)	487 (15)	597 (22.3)
	Moderate	1314 (22.2)	725 (22.3)	589 (22)
	Moderately severe	938 (15.8)	565 (17.4)	373 (13.9)
	Severe	871 (14.7)	616 (19)	255 (9.5)
Major Depressive Symptoms				
	No	4117 (69.5)	2065 (63.6)	2052 (76.6)
	Yes	1809 (30.5)	1181 (36.4)	628 (23.4)

Notes

^$^Other gender identity included intersex, Two-spirit (only for Canada), and other gender identity with an open-text box.

^£^Other sexual minority included gay/homosexual, lesbian, asexual, pansexual, queer, Two-spirit (only for Canada) and other sexual identity with an open-text box.

^§^Participants who selected any ethno-racial identity (one or more) other than white or Indigenous were classified as “racialized”. The category “non-racialized” includes young adults who selected “white” only and those who reported “white and Latino” or “white and Middle-Eastern” as per the definition in the Canadian Employment Equity Act. Indigenous category includes those who self-identify as First Nations, Métis, Inuk/Inuit descents.

^¶^Atlantic included the Canadian provinces of New Brunswick, Newfoundland and Labrador, Prince Edward Island, and Nova Scotia and Territories included Nunavut, Yukon, and the Northwest Territories.

^Nord Est (Grand-Est, Hauts-de-France, Bourgogne Franche-Comté), Sud Est (Auvergne-Rhône-Alpes, Provence-Alpes-Côte-d’Azur, Corse), Sud Ouest (Nouvelle Aquitaine, Occitanie), and Ouest (Bretagne, Centre Val-de-Loire, Pays de la Loire, Normandie).

**Fig 1:**
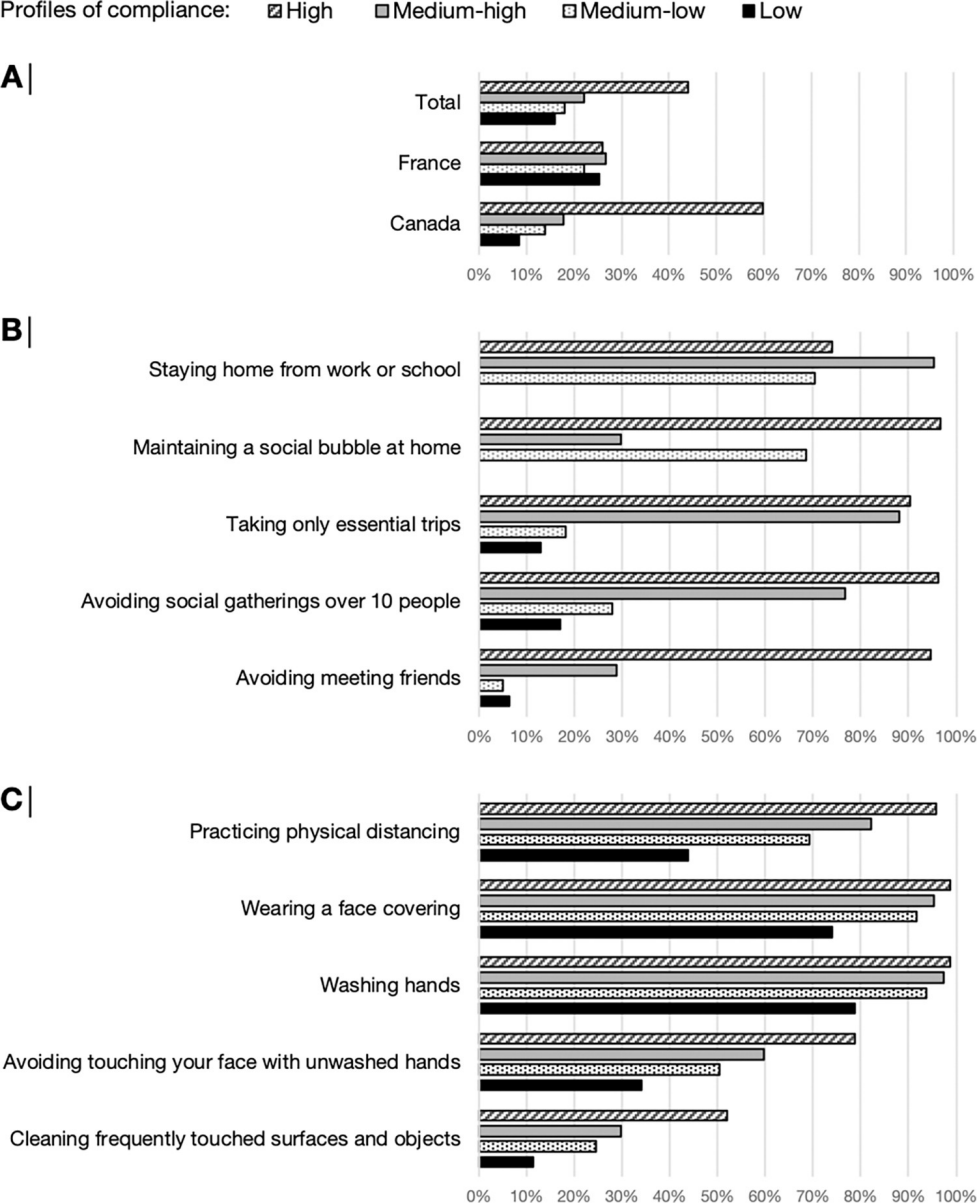
Description of the four profiles of compliance overall and by country (A), according to the COVID-19 preventive measures (B), and the health behaviours and hygiene practices (C).

Four COVID-19 compliance profiles were identified (Fig 1). In the profile with high compliance (n = 2521, 42.5%), more than 90% reported either maintaining a social bubble, taking only essential trips, and avoiding social gatherings and meeting friends; 72.9% reported staying at home. In the medium-high compliance profile (n = 1288, 21.7%), the vast majority reported staying at home (95.0%), taking only essential trips (88.2%), and avoiding social gatherings (76.4%). Only one third reported maintaining a social bubble (28.5%) and avoiding meeting friends (29.7%). Participants with medium-low compliance (n = 1071, 18.1%) reported maintaining a social bubble (68.4%) and staying at home (70.5%); fewer reported avoiding social gatherings (28.3%), taking only essential trips (17.3%), and avoiding meeting friends (5.2%). Among participants with low compliance (n = 1047, 17.7%), none reported staying at home or maintaining a social bubble and only a limited number reported avoiding meeting friends (6.2%), taking only essential trips (13.0%), or avoiding social gatherings (16.4%). As described in Fig 1, the COVID-19 compliance profiles were distributed differently by country. The majority of participants were highly compliant in Canada (56.2%) while a similar proportion of participants was found in each profile in France (high compliance: 26%, medium-high: 25.7%, medium-low: 21.4%, and low: 26.9%). Higher rates of compliance with COVID-19 health behaviors and hygiene practices were found in high compliance profiles compared to other profiles (see Fig 1C).

Table 2 presents the prevalence of major depressive symptoms by participants’ characteristics, and the level of compliance with COVID-19 preventive measures. In both countries, major depressive symptoms (Canada: 39.8%, France: 27.9%) were more prevalent among highly compliant participants than those who reported a low level of compliance (Canada: 32.7%, France: 17.7%).

**Table 2:** Prevalence of depression and associated factors, multivariable logistic regression, weighted and stratified by country.

		Canada (n = 3100)*				France (n = 2517)*			
		Prevalence of major depressive symptoms (n = 1110), n (row %)	AOR^*1*^	95% CI^*1*^	p-value	Prevalence of major depressive symptoms (n = 580), n (row %)	AOR^*1*^	95% CI^*1*^	p-value
**Socio-demographic characteristics**									
Age (years)									
	18–19	218 (45.5)	—	—		134 (31.1)	—	—	
	20–24	497 (37.7)	0.87	0.70, 1.07	0.2	260 (25.1)	0.98	0.78, 1.23	0.9
	25–29	395 (30.3)	**0.72**	**0.56, 0.93**	**0.012**	187 (17.7)	0.85	0.62, 1.16	0.3
Gender identity									
	Man	442 (30.7)	—	—		252 (19.8)	—	—	
	Woman	512 (37.2)	1.16	0.99, 1.35	0.065	281 (24.7)	**1.19**	**1.01, 1.40**	**0.041**
	Non-binary/other gender identity^$^	155 (55.4)	**1.65**	**1.26, 2.16**	**<0.001**	47 (44.2)	**2.35**	**1.67, 3.33**	**<0.001**
Sexual orientation									
	Straight/heterosexual	527 (29.9)	—	—		345 (20.1)	—	—	
	Bisexual	277 (44.9)	**1.52**	**1.27, 1.81**	**<0.001**	95 (28.6)	**1.23**	**1.01, 1.50**	**0.039**
	Other sexual minorities^£^	306 (42.5)	**1.43**	**1.19, 1.72**	**<0.001**	140 (30.2)	**1.38**	**1.13, 1.69**	**0.002**
Ethno-racial identity (only in Canada)^§^									
	Non-racialized	945 (34.8)	—	—					
	Indigenous	70 (49.1)	**1.45**	**1.03, 2.04**	**0.031**				
	Racialized, non-Indigenous	95 (38.9)	1.21	0.92, 1.58	0.2				
Descendants of immigrants (only in France)									
	No					477 (21.9)	—	—	
	Yes					103 (30.8)	**1.52**	**1.22, 1.91**	**<0.001**
Province or territory of residence (Canada)^¶^									
	Ontario	397 (35.2)	—	—					
	Atlantic	67 (34.4)	1.01	0.80, 1.28	>0.9				
	British Columbia/Territories	178 (39.5)	1.18	0.98, 1.42	0.084				
	Prairies	274 (43.7)	**1.4**	**1.15, 1.70**	**<0.001**				
	Quebec	195 (27.7)	0.86	0.70, 1.05	0.14				
Regions of residence (France)^^^						134 (21.7)	—	—	
	Ile-de-France					95 (19.1)	0.86	0.66, 1.11	0.2
	Nord-Est					111 (26.4)	1.24	0.97, 1.58	0.089
	Ouest					21 (28.8)	0.97	0.51, 1.83	>0.9
	Outre-mer					115 (22.6)	1.04	0.82, 1.32	0.7
	Sud-Est					104 (26.1)	1.21	0.96, 1.54	0.11
	Sud-Ouest								
Area of residence									
	Large urban centre	633 (33.5)	—	—		306 (23.6)	—	—	
	Medium or small city	477 (39.3)	**1.18**	**1.02, 1.37**	**0.026**	274 (22.5)	0.92	0.78, 1.08	0.3
Highest level of education									
	Some university	537 (34.1)	—	—		195 (21.5)	—	—	
	High school college	513 (42.1)	**1.38**	**1.18, 1.61**	**<0.001**	261 (30.1)	**1.34**	**1.10, 1.64**	**0.005**
	University graduate degree	60 (19.4)	**0.53**	**0.40, 0.70**	**<0.001**	124 (16.7)	0.82	0.64, 1.05	0.12
Employment status									
	Employed	391 (30.5)	—	—		127 (14.2)	—	—	
	Student	264 (39.1)	1.16	0.95, 1.42	0.13	257 (28.7)	**1.49**	**1.15, 1.92**	**0.002**
	Student and employed	287 (36.0)	1.12	0.92, 1.36	0.2	91 (21.8)	1.21	0.91, 1.60	0.2
	Unemployed	167 (48.5)	**1.58**	**1.25, 1.99**	**<0.001**	105 (34.1)	**2.42**	**1.83, 3.20**	**<0.001**
Living arrangements									
	With family members	440 (40.0)	—	—		198 (26.2)	—	—	
	Alone	169 (38.5)	**1.27**	**1.02, 1.58**	**0.033**	206 (25.9)	**1.33**	**1.09, 1.63**	**0.005**
	With partner	241 (29.6)	**0.83**	**0.68, 1.00**	**0.048**	102 (17.8)	0.79	0.62, 1.01	0.056
	With roomate/friends/other	260 (34.8)	0.85	0.71, 1.01	0.07	74 (18.9)	**0.69**	**0.54, 0.89**	**0.004**
**COVID-19-related concerns and experiences**									
Level of concern about the uncertainty of the future									
	Low	115 (14.0)	—	—		64 (10.0)	—	—	
	High	995 (43.6)	**4.06**	**3.34, 4.94**	**<0.001**	516 (27.6)	**2.75**	**2.17, 3.49**	**<0.001**
Level of concern for economy									
	Low	438 (33.2)	—	—		193 (21.0)	—	—	
	High	672 (37.7)	1.12	0.97, 1.29	0.12	387 (24.3)	1.02	0.86, 1.20	0.9
Being tested for COVID-19									
	No	699 (34.7)	—	—		319 (21.9)	—	—	
	Yes	411 (37.9)	**1.18**	**1.02, 1.37**	**0.022**	262 (24.6)	**1.23**	**1.06, 1.43**	**0.008**
Income loss									
	No	476 (30.8)	—	—		411 (21.8)	—	—	
	Yes	634 (40.7)	**1.16**	**1.01, 1.33**	**0.032**	170 (26.9)	**1.35**	**1.13, 1.61**	**0.001**
**Profiles of compliance with COVID-19 preventive measures**									
Level of compliance									
	High	693 (39.8)	—	—		182 (27.9)	—	—	
	Medium-high	188 (32.8)	**0.78**	**0.65, 0.93**	**0.008**	146 (22.3)	**0.77**	**0.63, 0.93**	**0.008**
	Medium-low	129 (27.0)	**0.58**	**0.48, 0.72**	**<0.001**	133 (24.9)	0.81	0.66, 1.01	0.056
	Low	101 (32.7)	**0.75**	**0.58, 0.98**	**0.033**	120 (17.7)	**0.6**	**0.48, 0.75**	**<0.001**

Notes

*Only participants with complete data for all covariates and explanatory factors were included in our regression models. After listwise deletion, the regression models consisted of a sub-sample with 3100 participants in Canada and 2517 in France. Among them, 1100 in Canada (35.5%) and 580 in France (23%) reported major depressive symptoms at the time of the survey.

^1^AOR adjusted odds ratios, CIs confidence intervals for the adjusted odds ratios; statistically significant associations (p < 0.05) are highlighted in bold.

^$^Other gender identity included intersex, Two-spirit (only for Canada), and other gender identity with an open-text box.

^£^Other sexual minority included gay/homosexual, lesbian, asexual, pansexual, queer, Two-spirit (only for Canada) and other sexual identity with an open-text box.

^§^Participants who selected any ethno-racial identity (one or more) other than white or Indigenous were classified as “visible minority”. The category “not visible minority” includes young adults who selected “white” only and those who reported “white and Latino” or “white and Middle-Eastern” as per the definition in the Canadian Employment Equity Act. Indigenous category includes those who self-identify as First Nations, Métis, Inuk/Inuit descents.

^¶^Atlantic included the Canadian provinces of New Brunswick, Newfoundland and Labrador, Prince Edward Island, and Nova Scotia and Territories included Nunavut, Yukon, and the Northwest Territories.

^Nord Est (Grand-Est, Hauts-de-France, Bourgogne Franche-Comté), Sud Est (Auvergne-Rhône-Alpes, Provence-Alpes-Côte-d’Azur, Corse), Sud Ouest (Nouvelle Aquitaine, Occitanie), and Ouest (Bretagne, Centre Val-de-Loire, Pays de la Loire, Normandie).

In multivariable analyses (Table 2), compared to the high compliance profile, participants in the low (Canada: Adjusted Odds Ratio (AOR) [95% Confidence Interval] 0.75 [0.58, 0.98], France: AOR 0.60 [0.46, 0.75]), in the medium-low (only in Canada: AOR 0.58 [0.48, 0.72]), and medium-high compliance profiles (Canada: AOR 0.78 [0.65, 0.93], France: AOR 0.77 [0.63, 0.93]) had lower odds of reporting major depressive symptoms. As shown in S2 Table, we found that the association between depression and COVID-19 compliance was significantly smaller in magnitude in Canada, as compared to France.

In both countries, young adults who identified as non-binary or with a gender identity other than man or woman (Canada: AOR 1.65 [1.26, 2.16], France: AOR 2.35 [1.67, 3.33]), and those who identified as bisexual (Canada: AOR 1.52 [1.27, 1.81], France: AOR 1.23 [1.01, 1.50]) or as a sexual minority (Canada: AOR 1.43 [1.19, 1.72], France: AOR 1.38 [1.13, 1.69]) had higher odds of reporting major depressive symptoms. Young adults who self-identified as Indigenous in Canada (AOR 1.45 [1.03, 2.04]), and descendants of immigrants in France (AOR 1.52 [1.22, 1.91]) had higher odds of reporting major depressive symptoms. Furthermore, being unemployed (Canada: AOR 1.58 [1.25, 1.99], France: AOR 2.42 [1.83, 3.20]) was associated with higher odds of reporting major depressive symptoms than those who were employed. Similar associations were found with having a high concern about the uncertainty of the future (Canada: AOR 4.06 [3.34, 4.94], France: AOR 2.75 [2.17, 3.49] or reporting income loss due to COVID-19 (Canada: AOR 1.16 [1.01, 1.33], France: AOR 1.35 [1.13, 1.61]).

## Discussion

One third of young adults who participated in our study reported major depressive symptoms. In both countries, participants with lower levels of compliance with COVID-19 preventive measures were less likely to experience major depressive symptoms. Similar high prevalence trends of moderate-to-severe depressive symptoms (PHQ-9 score≥10) were observed elsewhere among young adults in Canada (67%) [56] and among students in France (43%) [57]. In our study, we found a higher prevalence of major depressive symptoms in Canada compared to France (36.4% vs. 23.4%). This difference may be partially explained by socio-cultural differences with regards to expressing mental health concerns. For example, data collected before the pandemic indicated that Canadian adults were more likely to self-report experiences of emotional distress than French adults (27% vs. 12%) [58]. While significant efforts in mental health promotion and prevention interventions have been made in Canada in the last two decades [59], prior surveys in France documented persistent negative attitudes toward and perceptions of mental health issues (e.g., feelings of fear and shame, poor mental health literacy) before and during the pandemic [60, 61]. As such, there is a possibility that the terminology that we used in our questionnaire, including the term “mental health”, is viewed as a far more stigmatized condition in France versus Canada.

Our study also identifies an association between the level of COVID-19 preventive measure compliance and depressive symptoms. During the first year of the pandemic (March-December 2020), young adults who were highly compliant with socially-isolating preventive measures had less opportunities to engage with peers and to reduce time in isolation. These experiences may have been particularly challenging for young people ages 18–29, a period of the life course in which key social and professional developments are occurring. For example, several studies in the US identified multiple COVID-19-related stressors that may have contributed to the increased levels of depressive thoughts among students, such as decreased social interactions, disruptions to sleeping patterns, decline in physical activity, or spending more time on screens [62–65]. In parallel, several studies documented the negative impacts of the COVID-19 preventive measures on mental health services (e.g., disruptions, increased waiting times) which limited opportunities for young adults to find support to address depressive symptoms [66].

Furthermore, this association between compliance to COVID-19 preventive measures and depressive symptoms may partly be explained due to the differences in prevalence of depression between Canada and France, given that a higher proportion of participants with low levels of compliance were found in the French sample. This may be related to a higher prevalence of attitudes of distrust in the government actions and communication toward preventive measures in France compared to Canada, two contextual factors which are known to be critical determinants when designing and implementing campaign of prevention that requires a large-scale behavioral change [67]. In France, the communication about the COVID-19 pandemic and related public health measures has been mainly led by members of the Government of France with an independent committee of scientific experts, while in Canada, federal and provincial public health agencies integrated scientific advisors and experts as key representatives to promote the implementation of public health measures within each provincial and territorial jurisdiction [36, 68]. In a global survey assessing public attitudes towards governmental actions against the COVID-19 pandemic [69], the authors found lower levels of approval regarding the government communication about the pandemic (66% versus 81%) and the trust in governmental decisions (63% versus 77%) in France compared to Canada. Other contextual factors may explain the cross-country difference in COVID-19 compliance observed between Canada and France, including factors related to the implementation of socially restrictive COVID-19 measures (e.g., stringency, duration, enforcement) and the timing of the infection waves. For example, an analysis of a global database showed that the level of the strictness of COVID-19 governmental policies (i.e., assessed by a composite measure based on nine policy responses including school closures, workplace closures, and travel bans) was lower in France compared to Canada during the period of mid-June to mi-October 2020 [70, 71]. Further research is needed to investigate which and how contextual factors may play a role here.

Our findings also highlight that specific sub-groups of young adults reported higher rates of depressive symptoms. In both countries, sexual and gender minority youth were more likely to report major depressive symptoms compared to their heterosexual counterparts. Previous studies showed that sexual and gender minority youth experienced financial difficulties, limited access to gender-affirming resources, and increased experiences of discrimination, which therefore may increase the risk for depression [72–75]. Our analysis suggests that ethno-racial minorities had higher odds of experiencing major depressive symptoms. Similar high depression rates were found in diverse ethnic minority groups of students and adolescents [76, 77]. Previous research in the US reported that ethno-racial minorities have experienced several forms of discrimination during the pandemic [78, 79]. Such findings reinforce the need to strengthen access to mental health services for racialized youth. Depression was also more prevalent among students (only in France) and unemployed youth, among those who reported concerns about the uncertainty of the future, and among those who lost income due to COVID-19 (in both countries). As described in previous surveys [80, 81], the pandemic and related public health measures have created unprecedented conditions of stress and uncertainty regarding academic success, job opportunities, and future careers, that may explain these findings. Participants who had been tested for COVID-19 had higher risk for depression, a result in line with previous studies showing association between depression and COVID-19 contact, symptoms or diagnosis [50, 82].

### Strengths and limitations

Our study was conducted among a large and diverse sample of young adults, which allowed us to compare some population characteristics (e.g., men vs non-binary, unemployed vs employed) that have been rarely explored in previous COVID-19 studies among young adults. Our findings enabled us to further discuss the contextual and social-cultural factors that may have influenced the prevalence of depressive symptoms and the level of compliance in two high-income countries significantly impacted by the pandemic.

There are also several limitations. First, we used a non-probability sampling design and most participants were recruited via advertisements on social media. To reduce this bias, we then conducted analyses in each country on a sample that was weighted by age, gender, and province/region of residence. While this weighting procedure enables us to present greater extrapolation of our findings to the Canadian and French young adults’ population, our weighted samples are still not fully representative of the young adult population in Canada and France, which limits our ability to generalize these findings to all young adults living in both countries. Furthermore, our recruitment process might have led to the self-selection of respondents more concerned with COVID-19 than the general population. Second, our cross-sectional design does not allow us to clarify underlying causal mechanisms onto the association between compliance and depression. Third, we did not assess the participants’ history of mental health diagnostics.

### Public health implications

These findings provide critical insights into the association of socially-isolating public health measures and depressive symptoms among young adults. The implementation of such measures should be coupled with mental health support interventions to address the timely mental health needs of young adults, with recognition that those who are highly compliant with these measures may be more likely to experience depression. These kinds of interventions should be designed in a way to support the mental health needs of young adults who are structurally disadvantaged (e.g., unemployed, sexual and gender minority, ethno-racial minorities), as these are the mostly likely to be negatively impacted by public health restrictions.

## Conclusions

Our findings showed that young adults were engaged in COVID-19 preventive behaviors and also experienced high levels of depressive symptoms in Canada and France. Those who were more likely to be compliant with COVID-19 preventive measures reported higher prevalence of depressive symptoms. As such, this study provides new evidence in favour of the development and promotion of strategies that enhance access of young adults to equitable and non-judgemental mental health services. This is especially important for young adults who experienced various forms of social exclusion (e.g., poverty, racism, homophobia) during the COVID-19 pandemic.

## Supporting information

S1 Table Characteristics of the study population according to profiles of compliance with COVID-19 preventive measures. (DOCX)

S2 Table Cross-country difference in the magnitude of the association between depression and COVID-19 compliance profiles: Results of the Fairchild test. (DOCX)

S3 Table Comparative analysis of the sociodemographic characteristics between the study FOCUS participants included and participants excluded. (DOCX)
