# Supplementary material for: Association between level of compliance with COVID-19 public health measures and depressive symptoms: A cross-sectional survey of young adults in Canada and France
Source: PLoS One. 2023 Aug 2;18(8):e0289547. doi: 10.1371/journal.pone.0289547 (PMC10395933; doi:10.1371/journal.pone.0289547)
Supplement: S1 Table — (DOCX) [file pone.0289547.s001.docx]

S1 Table. Characteristics of the study population according to profiles of compliance with COVID-19 preventive measures.

|  |  | **Weighted** | | |  |  |  |  |  |  |  |  |  |  |
| --- | --- | --- | --- | --- | --- | --- | --- | --- | --- | --- | --- | --- | --- | --- |
|  |  | **Canada, n (column %)** | **Profiles of compliance with COVID-19 preventive measures, n (row %)** | | | |  |  | **France, n (column %)** | **Profiles of compliance with COVID-19 preventive measures, n (row %)** | | | |  |
|  |  | **Total,** | **Low** | **Medium-low** | **Medium-high** | **High** | **p-value*^1^*** |  | **Total** | **Low** | **Medium-low** | **Medium-high** | **High** | **p-value*^1^*** |
| **All participants** | | **3246 (100)** | **326 (10)** | **498 (15.3)** | **598 (18.4)** | **1824 (56.2)** |  |  | **2680 (100)** | **721 (26.9)** | **573 (21.4)** | **690 (25.7)** | **697 (26)** | |
| **Socio-demographic characteristics** | | |  |  |  |  |  |  |  |  |  |  |  |  |
| Age (years) | |  |  |  |  |  | <0.001 |  |  |  |  |  |  | <0.001 |
|  | 18-19 | 501 (15.4) | 12.6 | 25.1 | 16.4 | 13.0 |  |  | 459 (17.1) | 14.7 | 20.9 | 18.7 | 15.1 |  |
|  | 20-24 | 1379 (42.5) | 37.1 | 42.0 | 42.6 | 43.6 |  |  | 1101 (41.1) | 38.0 | 41.0 | 41.9 | 43.5 |  |
|  | 25-29 | 1366 (42.1) | 50.6 | 33.1 | 41.1 | 43.4 |  |  | 1120 (41.8) | 47.4 | 38.0 | 39.4 | 41.5 |  |
| Gender identity | |  |  |  |  |  | <0.001 |  |  |  |  |  |  | <0.001 |
|  | Man | 1547 (47.7) | 69.6 | 63.5 | 49.0 | 39.0 |  |  | 1321 (49.3) | 37.7 | 48.2 | 51.0 | 48.4 |  |
|  | Woman | 1411 (43.5) | 28.2 | 33.1 | 41.3 | 49.7 |  |  | 1238 (46.2) | 59.4 | 46.8 | 45.2 | 44.9 |  |
|  | Non-binary/other gender identity^$^ | 288 (8.9) | 2.1 | 3.4 | 9.7 | 11.3 |  |  | 121 (4.5) | 2.9 | 5.1 | 3.6 | 6.7 |  |
| Sexual orientation | |  |  |  |  |  | <0.001 |  |  |  |  |  |  | <0.001 |
|  | Straight/heterosexual | 1817 (56) | 74.8 | 67.1 | 56.5 | 49.4 |  |  | 1783 (66.5) | 74.5 | 63.7 | 64.1 | 63.1 |  |
|  | Bisexual | 617 (19) | 10.4 | 12.2 | 19.6 | 22.2 |  |  | 345 (12.9) | 10.4 | 12.2 | 15.1 | 13.8 |  |
|  | Other sexual minorities^£^ | 733 (22.6) | 12.9 | 18.3 | 21.9 | 25.8 |  |  | 478 (17.8) | 12.5 | 20.6 | 18.6 | 20.4 |  |
|  | Prefer not to say | 78 (2.4) | 1.8 | 2.4 | 2.0 | 2.6 |  |  | 73 (2.7) | 2.6 | 3.5 | 2.2 | 2.7 |  |
| Ethno-racial identity (only in Canada)^§^ | | |  |  |  |  | 0.006 |  |  |  |  |  |  |  |
|  | Non-racialized | 2821 (86.9) | 87.4 | 89.2 | 84.1 | 87.1 |  |  | _ |  |  |  |  |  |
|  | Indigenous | 149 (4.6) | 3.1 | 4.4 | 4.0 | 5.1 |  |  | _ |  |  |  |  |  |
|  | Racialized, non-Indigenous | 276 (8.5) | 9.5 | 6.4 | 11.7 | 7.8 |  |  | _ |  |  |  |  |  |
| Descendants of immigrants (only in France) | | |  |  |  |  |  |  |  |  |  |  |  | 0.12 |
|  | No | _ |  |  |  |  |  |  | 2307 (86.1) | 87.1 | 87.1 | 84.9 | 85.2 |  |
|  | Yes | _ |  |  |  |  |  |  | 353 (13.2) | 11.9 | 11.7 | 14.5 | 14.3 |  |
|  | Prefer not to say | _ |  |  |  |  |  |  | 20 (0.7) | 1.1 | 1.0 | 0.6 | 0.3 |  |
| Province or territory of residence (Canada)^¶^ | | |  |  |  |  | <0.001 |  |  |  |  |  |  |  |
|  | Ontario | 1174 (36.2) | 35.6 | 32.5 | 36.6 | 37.2 |  |  | _ |  |  |  |  |  |
|  | Atlantic | 204 (6.3) | 8.6 | 6.6 | 7.2 | 5.5 |  |  | _ |  |  |  |  |  |
|  | British Columbia/Territories | 472 (14.5) | 12.6 | 11.2 | 10.5 | 17.1 |  |  | _ |  |  |  |  |  |
|  | Prairies | 659 (20.3) | 24.8 | 18.7 | 17.4 | 20.9 |  |  | _ |  |  |  |  |  |
|  | Quebec | 737 (22.7) | 18.7 | 30.9 | 28.3 | 19.4 |  |  | _ |  |  |  |  |  |
| Regions of residence (France)^^^ | |  |  |  |  |  |  |  |  |  |  |  |  | <0.001 |
|  | Ile-de-France | _ |  |  |  |  |  |  | 658 (24.6) | 21.6 | 25.5 | 28.1 | 23.2 |  |
|  | Nord-Est | _ |  |  |  |  |  |  | 529 (19.7) | 20.9 | 16.4 | 17.4 | 23.5 |  |
|  | Ouest | _ |  |  |  |  |  |  | 450 (16.8) | 16.8 | 18.8 | 16.8 | 14.9 |  |
|  | Outre-mer | _ |  |  |  |  |  |  | 76 (2.8) | 2.1 | 1.6 | 2.5 | 4.9 |  |
|  | Sud-Est | _ |  |  |  |  |  |  | 542 (20.2) | 21.2 | 18.8 | 23.2 | 17.4 |  |
|  | Sud-Ouest | _ |  |  |  |  |  |  | 426 (15.9) | 17.3 | 18.8 | 12.0 | 15.9 |  |
| Area of residence | |  |  |  |  |  | 0.007 |  |  |  |  |  |  | <0.001 |
|  | Large urban centre | 1963 (60.5) | 56.1 | 56.0 | 62.9 | 61.7 |  |  | 1368 (51) | 46.6 | 54.5 | 54.6 | 49.4 |  |
|  | Medium or small city | 1283 (39.5) | 43.9 | 44.0 | 37.1 | 38.3 |  |  | 1312 (49) | 53.4 | 45.5 | 45.4 | 50.6 |  |
| Highest level of education | |  |  |  |  |  | <0.001 |  |  |  |  |  |  | 0.001 |
|  | High school college | 1285 (39.6) | 55.8 | 43.2 | 37.1 | 36.5 |  |  | 924 (34.5) | 34.3 | 38.9 | 31.4 | 34.0 |  |
|  | Some university | 1628 (50.2) | 36.8 | 48.4 | 51.3 | 52.7 |  |  | 967 (36.1) | 38.8 | 32.5 | 35.2 | 37.0 |  |
|  | University graduate degree | 326 (10) | 7.7 | 7.8 | 11.4 | 10.6 |  |  | 779 (29.1) | 26.8 | 27.9 | 32.6 | 28.7 |  |
|  | Missing data | 7 (0.2) | 0.0 | 0.6 | 0.2 | 0.2 |  |  | 10 (0.4) | 0.1 | 0.5 | 0.7 | 0.1 |  |
| Employment status | |  |  |  |  |  | <0.001 |  |  |  |  |  |  | <0.001 |
|  | Employed | 1323 (40.8) | 49.4 | 37.1 | 34.9 | 42.1 |  |  | 939 (35) | 42.0 | 29.1 | 33.6 | 34.0 |  |
|  | Student | 706 (21.7) | 10.7 | 22.1 | 28.9 | 21.2 |  |  | 945 (35.3) | 27.6 | 40.7 | 41.2 | 32.7 |  |
|  | Student and employed | 823 (25.4) | 23.0 | 30.5 | 25.4 | 24.3 |  |  | 455 (17) | 14.8 | 20.1 | 15.9 | 17.6 |  |
|  | Unemployed | 361 (11.1) | 15.3 | 9.8 | 10.0 | 11.1 |  |  | 326 (12.2) | 15.0 | 9.6 | 8.8 | 14.8 |  |
|  | Missing data | 33 (1) | 1.5 | 0.2 | 0.5 | 1.3 |  |  | 14 (0.5) | 0.6 | 0.3 | 0.3 | 0.9 |  |
| Living arrangements | |  |  |  |  |  | <0.001 |  |  |  |  |  |  | <0.001 |
|  | Alone | 455 (14) | 20.2 | 14.9 | 13.0 | 13.0 |  |  | 854 (31.9) | 35.5 | 31.1 | 32.2 | 28.4 |  |
|  | With family members | 1164 (35.9) | 29.8 | 41.0 | 39.0 | 34.5 |  |  | 809 (30.2) | 28.8 | 31.1 | 29.3 | 31.9 |  |
|  | With partner | 845 (26) | 23.0 | 22.3 | 22.2 | 28.9 |  |  | 600 (22.4) | 19.3 | 20.8 | 22.5 | 26.8 |  |
|  | With roomate/friends/other | 782 (24.1) | 27.0 | 21.9 | 25.8 | 23.6 |  |  | 414 (15.4) | 16.4 | 16.9 | 16.2 | 12.5 |  |
|  | Missing data | _ |  |  |  |  |  |  | 3 (0.1) | 0.1 | 0.0 | 0.0 | 0.3 |  |
| **COVID-19-related concerns and experiences** | | | |  |  |  |  |  |  |  |  |  |  |  |
| Level of concern about the uncertainty of the future | | | |  |  |  | 0.017 |  |  |  |  |  |  | 0.042 |
|  | Low | 844 (26) | 22.7 | 30.3 | 27.1 | 25.1 |  |  | 687 (25.6) | 28.4 | 24.6 | 24.3 | 24.8 |  |
|  | High | 2386 (73.5) | 76.1 | 69.5 | 72.2 | 74.5 |  |  | 1978 (73.8) | 71.2 | 75.0 | 75.1 | 74.0 |  |
|  | I don't know | 16 (0.5) | 1.2 | 0.2 | 0.5 | 0.4 |  |  | 12 (0.4) | 0.3 | 0.2 | 0.6 | 0.7 |  |
|  | Missing data | _ |  |  |  |  |  |  | 4 (0.1) | 0.1 | 0.0 | 0.0 | 0.3 |  |
| Level of concern for economy | |  |  |  |  |  | <0.001 |  |  |  |  |  |  | 0.088 |
|  | Low | 1360 (41.9) | 23.0 | 36.3 | 43.5 | 46.3 |  |  | 980 (36.6) | 38.1 | 35.6 | 36.5 | 35.7 |  |
|  | High | 1857 (57.2) | 76.4 | 62.4 | 55.4 | 53.0 |  |  | 1686 (62.9) | 61.0 | 63.7 | 63.2 | 64.0 |  |
|  | I don't know | 29 (0.9) | 0.6 | 1.2 | 1.3 | 0.8 |  |  | 12 (0.4) | 0.8 | 0.5 | 0.1 | 0.3 |  |
|  | Missing data | _ |  |  |  |  |  |  | 2 (0.1) | 0.0 | 0.2 | 0.1 | 0.0 |  |
| Being tested for COVID-19 | |  |  |  |  |  | <0.001 |  |  |  |  |  |  | 0.001 |
|  | No | 2112 (65.1) | 76.1 | 66.1 | 66.1 | 62.6 |  |  | 1547 (57.7) | 61.9 | 56.0 | 58.4 | 54.2 |  |
|  | Yes | 1124 (34.6) | 23.6 | 33.9 | 33.3 | 37.2 |  |  | 1124 (41.9) | 37.7 | 43.8 | 41.0 | 45.5 |  |
|  | Missing data | 9 (0.3) | 0.3 | 0.2 | 0.7 | 0.2 |  |  | 9 (0.3) | 0.4 | 0.0 | 0.6 | 0.3 |  |
| Income loss | |  |  |  |  |  | 0.9 |  |  |  |  |  |  | <0.001 |
|  | No | 1610 (49.6) | 47.9 | 49.4 | 50.3 | 49.8 |  |  | 2002 (74.7) | 70.6 | 74.0 | 78.0 | 76.3 |  |
|  | Yes | 1636 (50.4) | 52.1 | 50.6 | 49.7 | 50.2 |  |  | 678 (25.3) | 29.4 | 26.0 | 22.0 | 23.7 |  |
| **Mental health** | |  |  |  |  |  |  |  |  |  |  |  |  |  |
| Depressive symptoms (PHQ-9) | |  |  |  |  |  | <0.001 |  |  |  |  |  |  | <0.001 |
|  | Mild | 853 (26.3) | 23.0 | 29.3 | 28.9 | 25.2 |  |  | 866 (32.3) | 34.7 | 32.5 | 35.7 | 26.5 |  |
|  | Minimal | 487 (15) | 24.5 | 21.7 | 13.0 | 12.1 |  |  | 597 (22.3) | 28.2 | 18.0 | 22.5 | 19.5 |  |
|  | Moderate | 725 (22.3) | 19.9 | 20.7 | 23.4 | 22.9 |  |  | 589 (22) | 19.6 | 24.4 | 19.3 | 25.0 |  |
|  | Moderately severe | 565 (17.4) | 14.4 | 16.3 | 15.7 | 18.8 |  |  | 373 (13.9) | 11.8 | 14.8 | 12.8 | 16.5 |  |
|  | Severe | 616 (19) | 17.8 | 12.0 | 18.9 | 21.1 |  |  | 255 (9.5) | 5.8 | 10.1 | 9.9 | 12.6 |  |
| Major Depressive Symptoms | | |  |  |  |  | <0.001 |  |  |  |  |  |  | <0.001 |
|  | No | 2065 (63.6) | 67.8 | 71.7 | 65.4 | 60.1 |  |  | 2052 (76.6) | 82.4 | 75.0 | 77.4 | 71.0 |  |
|  | Yes | 1181 (36.4) | 32.2 | 28.3 | 34.6 | 39.9 |  |  | 628 (23.4) | 17.6 | 25.0 | 22.6 | 29.0 |  |

*Notes: ^1^p-values were calculated using chi-squared test with Rao & Scott's second-order correction*

*^$^Other gender identity included intersex, Two-spirit (only for Canada), and other gender identity with an open-text box.*

*^£^Other sexual minority included gay/homosexual, lesbian, asexual, pansexual, queer, Two-spirit (only for Canada) and other sexual identity with an open-text box.*

^§^*Participants who selected any ethno-racial identity (one or more) other than white or Indigenous were classified as “visible minority”. The category “not visible minority” includes young adults who selected “white” only and those who reported “white and Latino” or “white and Middle-Eastern” as per the definition in the Canadian Employment Equity Act. Indigenous category includes those who self-identify as First Nations, Métis, Inuk/Inuit descents.*

*^¶^Atlantic included the Canadian provinces of New Brunswick, Newfoundland and Labrador, Prince Edward Island, and Nova Scotia and Territories included Nunavut, Yukon, and the Northwest Territories.*

*^Nord Est (Grand-Est, Hauts-de-France, Bourgogne Franche-Comté), Sud Est (Auvergne-Rhône-Alpes, Provence-Alpes-Côte-d’Azur, Corse), Sud Ouest (Nouvelle Aquitaine, Occitanie), and Ouest (Bretagne, Centre Val-de-Loire, Pays de la Loire, Normandie).*
