# Supplementary material for: Association between level of compliance with COVID-19 public health measures and depressive symptoms: A cross-sectional survey of young adults in Canada and France
Source: PLoS One. 2023 Aug 2;18(8):e0289547. doi: 10.1371/journal.pone.0289547 (PMC10395933; doi:10.1371/journal.pone.0289547)
Supplement: S2 Table — (DOCX) [file pone.0289547.s002.docx]

S2 Table: Cross-country difference in the magnitude of the association between depression and COVID-19 compliance profiles: results of the Fairchild test.

|  | **Canada** |  | **France** |  |  | **Difference between Canada and France** | | |
| --- | --- | --- | --- | --- | --- | --- | --- | --- |
|  | **Coefficient** | **Standard Error** | **Coefficient** | **Standard Error** |  | **Difference between coefficients** | **Low** | **Up** |
| **Profiles of level of compliance** |  |  |  |  |  |  |  |  |
| Low | -0.28139 | 0.13201 | -0.50926 | 0.11556 |  | 0.22787 | -0.1160011 | 0.5717411 |
| Medium-low | -0.53762 | 0.10519 | -0.20573 | 0.10747 |  | **-0.33189** | **-0.6266387** | **-0.0371413** |
| Medium-high | -0.25188 | 0.09416 | -0.26743 | 0.1012 |  | 0.01555 | -0.2553809 | 0.2864809 |

*Notes: Statistically significant associations (p < 0.05) are highlighted in bold.*
