# Supplementary material for: Association between level of compliance with COVID-19 public health measures and depressive symptoms: A cross-sectional survey of young adults in Canada and France
Source: PLoS One. 2023 Aug 2;18(8):e0289547. doi: 10.1371/journal.pone.0289547 (PMC10395933; doi:10.1371/journal.pone.0289547)
Supplement: S3 Table — (DOCX) [file pone.0289547.s003.docx]

**S3 Table. Comparative analysis of the sociodemographic characteristics between the study FOCUS participants included and participants excluded.**

|  |  | **Non-weighted,**  **n (column %)** | |  | **Non-weighted,**  **n (column %)** | |  |
| --- | --- | --- | --- | --- | --- | --- | --- |
|  |  | **Participants included** | **Participants excluded** | **p-value** | **Participants with missing compliance data** | **Participants with missing mental health data** | **p-value** |
| **All participants** | | **5926 (100)** | **1619 (27.3)** |  | **383 (5.1)** | **1236 (16.4)** |  |
| Country of residence | |  |  | <0.001 |  |  | <0.001 |
|  | Canada | 3246 (54.8) | 630 (38.9) |  | 134 (35) | 496 (40.1) |  |
|  | France | 2680 (45.2) | 989 (61.1) |  | 249 (65) | 740 (59.9) |  |
| Age (years) | |  |  | <0.001 |  |  | <0.001 |
|  | 18-19 | 1060 (17.9) | 435 (26.9) |  | 91 (23.8) | 344 (27.8) |  |
|  | 20-24 | 2781 (46.9) | 745 (46) |  | 177 (46.2) | 568 (46) |  |
|  | 25-29 | 2085 (35.2) | 439 (27.1) |  | 115 (30) | 324 (26.2) |  |
| Gender identity | |  |  | <0.001 |  |  | <0.001 |
|  | Woman | 3780 (63.8) | 918 (56.7) |  | 201 (52.5) | 717 (58) |  |
|  | Man | 1747 (29.5) | 621 (38.4) |  | 163 (42.6) | 458 (37.1) |  |
|  | Non-binary/other gender identity^$^ | 399 (6.7) | 80 (4.9) |  | 19 (5) | 61 (4.9) |  |
| Sexual orientation | |  |  | <0.001 |  |  | <0.001 |
|  | Straight/heterosexual | 3524 (59.5) | 1060 (65.5) |  | 245 (64) | 815 (65.9) |  |
|  | Bisexual | 1116 (18.8) | 245 (15.1) |  | 62 (16.2) | 183 (14.8) |  |
|  | Other sexual minorities | 1134 (19.1) | 251 (15.5) |  | 58 (15.1) | 193 (15.6) |  |
|  | Prefer not to say | 152 (2.6) | 0 (0) |  | 0 (0) | 0 (0) |  |
|  | Missing data | 0 (0) | 63 (3.9) |  | 18 (4.7) | 45 (3.6) |  |
| Ethno-racial identity (only in Canada) | |  |  | 0.2 |  |  | 0.3 |
|  | Non-racialized | 2858 (88) | 555 (88.1) |  | 117 (87.3) | 438 (88.3) |  |
|  | Indigenous | 157 (4.8) | 22 (3.5) |  | 3 (2.2) | 19 (3.8) |  |
|  | Racialized, non-Indigenous | 231 (7.1) | 53 (8.4) |  | 14 (10.4) | 39 (7.9) |  |
| Descendants of immigrants (only in France) | |  |  | <0.001 |  |  | <0.001* |
|  | No | 2343 (87.4) | 967 (97.8) |  | 248 (99.6) | 719 (97.2) |  |
|  | Yes | 319 (11.9) | 19 (1.9) |  | 1 (0.4) | 18 (2.4) |  |
|  | Prefer not to say | 18 (0.7) | 3 (0.3) |  | 0 (0) | 3 (0.4) |  |
| Province or territory of residence (Canada) | |  |  | 0.2 |  |  | 0.031 |
|  | Ontario | 692 (21.3) | 139 (22.1) |  | 34 (25.4) | 105 (21.2) |  |
|  | Atlantic | 386 (11.9) | 96 (15.2) |  | 20 (14.9) | 76 (15.3) |  |
|  | British Columbia/Territories | 741 (22.8) | 137 (21.7) |  | 23 (17.2) | 114 (23) |  |
|  | Prairies | 690 (21.3) | 125 (19.8) |  | 18 (13.4) | 107 (21.6) |  |
|  | Quebec | 737 (22.7) | 133 (21.1) |  | 39 (29.1) | 94 (19) |  |
| Regions of residence (France) | |  |  | 0.013 |  |  | 0.046* |
|  | Ile-de-France | 565 (21.1) | 189 (19.1) |  | 55 (22.1) | 134 (18.1) |  |
|  | Nord-Est | 469 (17.5) | 175 (17.7) |  | 51 (20.5) | 124 (16.8) |  |
|  | Ouest | 482 (18) | 215 (21.7) |  | 45 (18.1) | 170 (23) |  |
|  | Outre-mer | 31 (1.2) | 15 (1.5) |  | 5 (2) | 10 (1.4) |  |
|  | Sud-Est | 611 (22.8) | 222 (22.4) |  | 53 (21.3) | 169 (22.8) |  |
|  | Sud-Ouest | 522 (19.5) | 170 (17.2) |  | 38 (15.3) | 132 (17.8) |  |
|  | Missing data | 0 (0) | 3 (0.3) |  | 2 (0.8) | 1 (0.1) |  |
| Area of residence | |  |  | <0.001 |  |  | <0.001 |
|  | Large urban centre | 3150 (53.2) | 753 (46.5) |  | 181 (47.3) | 572 (46.3) |  |
|  | Medium or small city | 2776 (46.8) | 866 (53.5) |  | 202 (52.7) | 664 (53.7) |  |
| Highest level of education | |  |  | <0.001 |  |  | <0.001 |
|  | High school college | 2264 (38.2) | 821 (50.7) |  | 173 (45.2) | 648 (52.4) |  |
|  | Some university | 2690 (45.4) | 585 (36.1) |  | 145 (37.9) | 440 (35.6) |  |
|  | University graduate degree | 956 (16.1) | 197 (12.2) |  | 60 (15.7) | 137 (11.1) |  |
|  | Missing data | 16 (0.3) | 16 (1) |  | 5 (1.3) | 11 (0.9) |  |
| Employment status | |  |  | 0.027 |  |  | 0.029* |
|  | Employed | 2073 (35) | 233 (14.4) |  | 1 (0.3) | 232 (18.8) |  |
|  | Student | 1830 (30.9) | 244 (15.1) |  | 0 (0) | 244 (19.7) |  |
|  | Student and employed | 1363 (23) | 137 (8.5) |  | 0 (0) | 137 (11.1) |  |
|  | Unemployed | 618 (10.4) | 87 (5.4) |  | 1 (0.3) | 86 (7) |  |
|  | Missing data | 42 (0.7) | 918 (56.7) |  | 381 (99.5) | 537 (43.4) |  |
| Living arrangements | |  |  | <0.001 |  |  | <0.001* |
|  | Alone | 1268 (21.4) | 147 (9.1) |  | 1 (0.3) | 146 (11.8) |  |
|  | With family members | 1971 (33.3) | 261 (16.1) |  | 1 (0.3) | 260 (21) |  |
|  | With partner | 1497 (25.3) | 112 (6.9) |  | 0 (0) | 112 (9.1) |  |
|  | With roomate/friends/other | 1187 (20) | 133 (8.2) |  | 1 (0.3) | 132 (10.7) |  |
|  | Missing data | 3 (0.1) | 966 (59.7) |  | 380 (99.2) | 586 (47.4) |  |
| *Notes: The p-values were calculated from Pearson's Chi-squared test and Fisher's exact test.*  **The p-values were obtained by examining only the difference between the included participants and those who had incomplete data for mental health.*  *$Other gender identity included intersex, Two-spirit (only for Canada), and other gender identity with an open-text box.*  *£Other sexual minority included gay/homosexual, lesbian, asexual, pansexual, queer, Two-spirit (only for Canada) and other sexual identity with an open- text box.*  *§Participants who selected any ethno-racial identity (one or more) other than white or Indigenous were classified as “racialized”. The category “non-racialized” includes young adults who selected “white” only and those who reported “white and Latino” or “white and Middle-Eastern” as per the definition in the Canadian Employment Equity Act. Indigenous category includes those who self-identify as First Nations, Métis, Inuk/Inuit descents.*  *¶Atlantic included the Canadian provinces of New Brunswick, Newfoundland and Labrador, Prince Edward Island, and Nova Scotia and Territories included Nunavut, Yukon, and the Northwest Territories.*  *^Nord Est (Grand-Est, Hauts-de-France, Bourgogne Franche-Comté), Sud Est (Auvergne-Rhône-Alpes, Provence-Alpes-Côte-d’Azur, Corse), Sud Ouest (Nouvelle Aquitaine, Occitanie), and Ouest (Bretagne, Centre Val-de-Loire, Pays de la Loire, Normandie).* | | | | | | | |
